# A comparison of the nationally important infection prevention and control documents in NHS England and NHS Scotland

**DOI:** 10.1177/1757177420971849

**Published:** 2020-11-23

**Authors:** Evonne T Curran, Emma Burnett, Jude Robinson, Heather Loveday

**Affiliations:** 1 Independent Infection Control Nurse Consultant; 2 University of Dundee, Dundee, UK; 3 Nottinghamshire Healthcare NHS Trust, Nottingham, UK; 4 University of West London, Brentford, Middlesex, UK

**Keywords:** National priority, infection control, quality improvement

## Abstract

**Background:**

The devolution of health to Scotland in 1999, led for the first time in the NHS, to different priorities and success indicators for infection prevention and control (IPC). This project sought to understand, compare and evaluate the national IPC priorities and available indicators of success.

**Aim:**

To identify the national IPC priorities alongside national indicators of success.

**Methods:**

Critical analysis of nationally produced documents and publicly available infection-related data up to March 2018.

**Findings:**

For both NHS Scotland and England the local and national IPC priorities are evidenced by: (1) people being cared for in an IPC-safe environment; (2) staff following IPC-safe procedures; and (3) organisations continuously striving not just to attain standards, but to improve on them. If national agencies that produce data were also charged with using a Continuous Quality Improvement (CQI) model, then there would be further opportunities to detect and improve on successes.

## Introduction

‘*The specific effects of devolution on health policy are clearly impossible to predict.*’

Colin Leys, *BMJ* 1999 p. 1155

In 1999, the UK government devolved the responsibility for health to national assemblies; this gave the then new Scottish Executive Health Department (SEHD) the capacity to take different paths from the NHS in England. In the early years after devolvement, NHS Scotland stayed as a mirror image of NHS England; however, over time, and in response to emerging infection control problems, this became less so. Although both health departments are still clearly influenced by each other, in some infection prevention and control (IPC) policy issues and in data presentations, their paths have diverged. By the time what can be described as the IPC’s lowest point—or nadir—was reached (around 2006), experienced as seemingly endless outbreaks of MRSA and *Clostridium difficile* infection (CDI) and with mortality from hospital-acquired infections (HAI) still rising, each nation was producing different solutions to the same problems. Curran (2021) discusses this IPC nadir in detail. Thus, these differences provide an opportunity to compare the separate national IPC documents to evaluate differing IPC priorities and any successes. As part of a wider research project, the aim of the present study was to identify national IPC priorities and the indicators of success in the UK and Ireland. This paper compares the national IPC priority documents, the IPC successes—as identified in national data and reports—for the acute services of NHS England and NHS Scotland (there being insufficient space to present all countries’ data in a single paper). The figures discussed in this paper are presented online at: **web link here**.

## Research questions

Question 1: What are the local and national infection prevention and control priorities?
Question 2: What are considered to be the indicators of success and how are they measured?

## Methods

Senior representatives from NHS England and NHS Scotland were asked to list their national IPC priority documents (Table 1). These documents were reviewed using a structured assessment form. For each document, the IPC topic areas were categorised (a topic was considered to be an IPC area of work such as ‘surveillance’, ‘decontamination’ or ‘use of invasive devices’) in order to identify how many topics were included in each priority document, how many were unique to that document and how many overlapped with other priority documents. A topic for which multiple priority documents provided instructions or standards could prove confusing for those trying to follow the instructions and those monitoring the people/services doing so. A model was selected to best depict the structure of each health service (Organisational Cybernetics Model). This model enables ‘a shared understanding of the organizational complexity’ to be communicated (Jackson, 2004, p.109). This model also shows the structure of each health service and how the relationships and feedback processes operate within the individual levels of each system. For example, Level I comprises the clinical microsystems that represent individual wards, theatres or outpatients and the people who staff them. All of Level I report to a higher level of management at Level II. Level VI represents the highest structure of the organisation, the government’s Departments of Health.

**Table 1.** IPC priority documents in NHS England and NHS Scotland.

NHS England	Topics	Unique	NHS Scotland	Topics	Unique
1 The Health and Social Care Act (Code of Practice) (2008 updated 2015) Dept of Health (England) (includes a list of 25 policies)	10	4	1 HAI Standards 2015 Healthcare Improvement Scotland	8	5
2 NICE. Infection Prevention and Control Quality Standard QS61 (2014)	6	1	2 National Infection Prevention and Control Manual from 2012 Health Protection Scotland	3	0
3 NICE. Healthcare-associated infections: prevention and guidance. Public Health Guideline 36 (2011)	11	2	3 HAI Surveillance as listed in DL (2015) 19 Lists all required screening, surveillance and reporting	10	8
4 NICE. Healthcare-associated infections Quality Standard 113 (2016)	4	1	4 National Support Framework CNO Support Algorithm	5	3
5 NHS England. CQUINs: Flu vaccination / Sepsis, Antibiotic assessment, Antibiotic Consumption	1	0	5 Scotland Health Facilities Notes (SHFN) 30 Manual Information for Design Estates and the IPCT HAI Scribe Implementation Strategy and Assessment Process 2015	2	2
6 NHS Improvement. Annual CDI Objective 2018–2019 (2017)	1	0			
7 NHS Improvement. Update on the reporting and monitoring arrangements and post-infection review process for MRSA blood stream infections (2018)	1	0			
8 NHS Improvement. Preventing healthcare associated Gram-negative bloodstream infections: an improvement resource (2017)	5	2			
9 Department of Health. Implementation of modified admission MRSA guidance NHS Screening (2014)	1	0			
	40	10		28	18
Unique topics (%)		25			64

These documents are referred to in the text as England or Scotland with their appropriate number.

The national priority documents were categorised by the goals/tasks to be achieved, the levels within the cybernetics model responsible for the goals/tasks and any specified monitoring authorities. Documents which had overlapping topic content, e.g. both specifying criteria to be attained in areas such as surveillance or environmental cleanliness, were then identified. A schematic was produced showing all the documents and where there was overlapping content.

Outcome indicator data were identified by retrieving national surveillance publications, reports from national scrutiny organisations, and from the departments of health and social care websites (up to March 2018).

From all the above data, a qualitative assessment was made to answer the research questions.

## Results

### Organisation variation

An Organisational Cybernetics Model for both NHS Scotland and NHS England were produced comprising six levels (Figures 1 and 2 for NHS Scotland and NHS England, respectively). For both England and Scotland, Level I consists of clinical microsystems (CMS), e.g. individual wards or departments that report to Level II, a unit/directorate management. Several Level II units report into a higher level of management (Level III) before subsequently reporting to a single Chief Executive Officer (CEO) at Level IV. The CEOs in Scotland report to a single board (Level V) before all boards report to Level VI, the (now titled) Scottish Government’s Health and Social Care Department. In England, the CEOs report to the Clinical Commissioning Groups (CCGs) who plan and commission services for their areas. All levels (I–VI) in both Scotland and England are supported by colleagues, organisations and the public at three separate levels.

The first, Support I, comprises service user monitoring. In Scotland, this is done formally alongside the Healthcare Environment Inspectorate (HEI). In England, Support I is provided by Patient-Led Assessments of the Care Environment (PLACE) monitoring with service users. Also included in Support I is independently and surveyed public feedback reporting on services received. Support II includes all the service facilitators, e.g. education and training, occupational health, estates management and the microbiology lab, who along with internal monitoring provide support to Levels I–V. The IPC Team (IPCT) is also part of Support II. The IPCT receives and provides information to Levels I–V. Support III includes the external support and monitoring provided by Health Protection Scotland (HPS), Health Facilities Scotland (HFS). the HEI and Healthcare Improvement Scotland (HIS). In England, Support III is provided by Public Health England (PHE), the Care Quality Commission (CQC) and NHS Improvement. Although the designs are very similar, with both having the six recursive layers, the overall size and variant structures (reporting and commissioning) illustrate the differences.

### IPC priority documents

The IPC priority documents nominated for NHS Scotland and NHS England are shown in Table 1. Although NHS Scotland listed only five documents, one (DL (2015) 19) specifies all required surveillance programmes and the required compliance. Similarly, the Code of Practice for NHS England lists 25 specific policies to be available within Trusts, along with 10 system specifications to ensure a safe IPC environment. The overlap in these documents can be seen in Figure 3 (Scotland) and Figure 4 (England), and Table 1. NHS Scotland have fewer individual topics than NHS England (28 vs. 40) and there is less overlap between the documents. Unique topics, those appearing in only one priority document, were identified in 25% of NHS England documents compared to 64% of those published by NHS Scotland. The rationale for multiple documents containing different criteria to be adhered to on similar topics is unstated. Apart from compliance with the Code of Practice in England being a legal requirement, the multiple documents with overlapping content results in—from an outside perspective, at least—difficulties for both those who must achieve and monitor adherence to the criteria.

### Responsibility for fulfilling the criteria in the priority documents

In NHS England and NHS Scotland, most of the topic criteria within the priority documents were tasked to Level I healthcare workers (as per the cybernetic models in Figures 1 and 2) at 26.7% and 24.1%, respectively, and to the IPCT at 35% and 41.2%, respectively.

### The national IPC priorities

From the reading of the IPC priority documents (Table 1), an assessment of the IPC topics and specifications was made for both NHS Scotland and NHS England. While the policies varied in complexity and overlapped in topics, they had similarities and both authorities aimed to:

- specify the requirements of an IPC safe environment;
- specify the IPC governance arrangements;
- specify how some IPC procedures are to be performed;
- set up external organisations to:
  - regulate and monitor healthcare environments, governance arrangements and various clinical procedures
  - publish reports of their findings so that the public are provided with evidence of IPC safety
- specify numerical targets or goals, the achievement of which would indicate whether the above arrangements were impacting on rates of infection;
- require organisations to undertake IPC safety within a quality improvement framework, i.e. continuously improving rates of HAI and continuously minimising risks;
- detect and respond to emerging threats.

Apart from the last criteria, these were new requirements before 2006.

For both national NHSs, several priority documents specify the need for continuous quality improvement (CQI) for Levels I–VI, e.g. priority documents: NHS England 2, 3, 4 and NHS Scotland 1 (Figures 3 and 4).

These results advocate ‘Continuous Quality Improvement’, which is defined as an effective, efficient method that aims is to continually improve the overall quality-related performance (Juran and Godfrey, 1998). This is different from quality assurance, which aims to demonstrate that the requirements for quality have been (and can be) achieved. The key difference is therefore between meeting a requirement and continually improving (Juran and Godfrey, 1998).

### Quantitative indicators of IPC success

The quantitative indicators, for which there are enough data to detect the presence, or absence of, successes are: surgical site infections (SSI); MRSA bacteraemia; and CDI. These data are collected through national surveillance programmes that specified the data to be collected, the definitions to be used and the time scales for data submission. These data are used to identify the priorities for prevention programmes and to guide investigations when data suggest significant variations. The significant declines in both MRSA bacteraemia and CDI were evident UK-wide from 2007. This analysis focuses on the trends from 2014 that can be used as current indicators of success. Having stated that Levels I–V of the organisational cybernetics model (Figures 1 and 2) are instructed to use a CQI model, national organisations producing accumulated data from health boards and Trusts are not. Where possible from the national data available in the public domain, statistical process control charts (SPCs) were produced (by ETC) in order to accurately describe the variation in the data as either natural or unnatural (Benneyan, 1998).

### Current MRSA bloodstream infections (MRSA BSI)

MRSA BSI data for NHS England have been published in spreadsheets, the format of which has been modified overtime (e.g. total reported, trust assigned, CCG assigned and/or third party assigned). However, all these spreadsheets have only 12 or 13 months of data per spreadsheet page. All spreadsheets used in this analysis were retrieved from the UK Government’s live and archive surveillance web pages (UK Government, 2018a). As there are only 13 data points on a spread sheet, it is neither possible to determine whether the data are in or out of statistical control (above or below the control limits) nor whether the within-limit criteria for being out of control are met. An SPC (produced by ETC, from the spreadsheets not shown) of 22 months of Trust-apportioned MRSA data (May 2016 to March 2018) shows the data to be currently in statistical control. The number of ‘trust-apportioned’ infections for January to March 2017 is at 100; for the same period in 2014, there were 106 (UK Government, 2018b). Of note, even though the results have currently plateaued, the infections reduced from the peaks of 2006 have been retained.

For Scotland, the number of MRSA BSI are no longer separately produced. These data are combined with MSSA BSI data, for which an ongoing increase is evident (HPS, 2017a, 2018a, 2018b). Thus, a previously achieved target and evidence of success in MRSA BSI reductions, has been negated by a missed target of MSSA reductions which may not itself be achievable. Data are presented quarterly, three months in arrears with a limit of 12 quarterly data points per spreadsheet. So, once again, within-limit out-of-control criteria cannot be assessed. Although SPC charts are produced in Scotland, they have no lower control limits and, as stated, insufficient data for a valid assessment of variation.

### Current situation with *Clostridium difficile* infection (CDI)

The same data issues arise with national CDI data, in that the data are presented in spreadsheets comprising just 12–13 months of data. These spreadsheets have also been modified overtime. All spreadsheets used are available from the UK Government’s live and archive web pages (UK Government, 2019).

For NHS England data, again spreadsheet presentations have been modified over time; however, all are set in a spreadsheet of 12–13 months of data. Again, the results are not presented to enable easy assessment of data variation. An SPC was produced for 24 months of trust-apportioned data, showing one out-of-control episode and the overall chart with wide monthly variation. Although the chart is currently in control, a warning limit was reached in March 2018. Declines in CDI have also plateaued—this despite the target remaining in place and financial penalties being applied for failing to achieve them in NHS England. No commentary could be found on the data published in these. A commentary which interprets both local data and national trends is important to ensure that the message being sent via graphical representation of the data is understood by those receiving it.

For NHS Scotland, the CDI data are presented within a spreadsheet with two tables comprising 12 points of data (four quarters of three years), for each NHS board (and Scotland as a whole) (HPS, 2018a, 2018b). The two tables are for ‘healthcare-associated’ (HA) and ‘community-associated’ (CA) infections. Alongside the tables in this supplementary data are two sets of charts (two for each HA and CA infections). Both sets of charts have the same title and neither states clearly what the data are, i.e. what the lines indicate. Their two references for web information lack specific guidance as to how to use these charts to detect statistical control for SAB or CDI (HPS, 2017b). A commentary is provided comparing current to past quarters – but true over-time variation comment (i.e. on 25 data points) is omitted.

### Surgical site infections as indicators of success

Surgical site infection (SSI) surveillance is the ongoing monitoring of surgical infection rates fed back to those involved in the operations (and their managers) and fed forward to national surveillance organisations. Feeding forward enables national organisations to compare data from individual centres and identify outliers where specialist assistance can be offered. This continuous quality improvement initiative was developed from initial work in America which identified that surveillance of SSI with feedback was effective at reducing rates of infection (Haley et al., 1985). National SSI surveillance programmes are ongoing in all countries in the UK. In 2004, surveillance of SSI in orthopaedic surgery became mandatory for all English NHS Trusts (PHE, 2013). This allows NHS Trusts to compare their rates of infection over time against a benchmark rate. NHS England has data over several years showing decreasing SSI rates with increased participation PHE (2017). Similarly, NHS Scotland publishes quarterly rates of SSI data showing sustained low rates of infection for mandatory procedures (HPS, 2018a, 2018b). These data can be used to indicate success but only for the procedures under surveillance.

### Qualitative assessments by external inspectors

The external monitoring authorities (CQC in England and the HEI in Scotland) produce reports of announced and unannounced assessments of the IPC healthcare environment: as seen, as reported by people they talk to and as reported by people through surveys. The CQC reports, which scrutinise governance, have resulted in improvements in ratings over time; indeed, the CQC report states ‘[we have] seen that it delivers improved care’ (CQC, undated). The publicity surrounding reports and the requirement to display CQC ratings informs Trust CEOs (Level IV) what they need to do to have a positive CQC indicator of success. PLACE assessments (NHS England), which involve the public in evaluations of visible cleanliness and condition and maintenance of the clinical environments, have yielded an indicator of success. For example, in 2017, the national average score for cleanliness was 98.4%, which was the highest since such measures began, the median score being 99.3% (PLACE, 2017). In Scotland, the HEI uses the criteria within the HAI Standards from Healthcare Improvement Scotland (2015) when inspecting. All reports are web-listed under the NHS Board so that anyone can see any hospital’s results (Healthcare Improvement Scotland, 2018). Publications of new standards in 2015 resulted in a change to the cumulative reporting, making it difficult to determine if there are continued ongoing improvements. Certainly, no observed/reported hazardous cleanliness issues appear to merit media reporting as there was during what was discussed earlier as the IPC nadir (Curran, 2021).

## Discussion

The aim of the present study was to evaluate national IPC priorities and indicators of success. The national IPC priorities are that people are cared for in an IPC-safe environment, using IPC-safe procedures. The indicators of success are that there is evidence from external reports of this being achieved and an absence of external reports of poorly managed risks in the media, e.g. large outbreaks. Also, there are national data indicating that numerical infection risks are low and staying low.

What the project also enabled was a comparison of the ways the NHS in England and NHS Scotland have diverged in their goals of achieving IPC safety and the opportunities this presents for improvement. Devolution, which began in 1999, has led to different priority documents that essentially seek the same goals. However, in NHS England, there are several documents that have multiple requirements for the same topic area, e.g. governance and surveillance (Figure 4). Also, whereas NHS Scotland (Figure 3) has updated documents and removed redundant ones (e.g. Code of Practice), NHS England has added new documents to existing ones so that several documents have specifications on the same topic. Therefore, from an operational perspective, it is easier to see from NHS Scotland’s priority documents precisely what external monitoring agencies should be assessing. The production of a national IPCM means that 14 NHS Boards are unburdened with producing and updating their own policies and thus have more time to focus on implementation. Conversely, the NHS in England requires all trusts to have and update 25 separate policies. For external monitors in Scotland, the focus can be solely on implementation, whereas for England, monitors should seek assurance that policies are present, up to date and being implemented. This is now being addressed in England. The periodic updating of the requirements of the IPC system in Scotland appears useful (e.g. DL (2015) 19). Furthermore, simplifying and reassessing the expectations of IPCTs periodically would reduce redundancy and free up time for emerging IPC challenges.

From the assessment of national surveillance reports, there appears to be a misalignment of purpose. Levels I–IV of healthcare organisations are tasked with undertaking their work in the context of CQI; however, those producing national data are not (Support III, i.e. PHE and HPS; Figures 1 and 2). This is evidenced by the data being utilised to compare rates rather than to detect and drive improvement, e.g. the HPS SPCs have insufficient data points and omit a lower control limit and are thus incapable of detecting improved performance. If Support III was charged with providing data back to Trusts/Boards using a CQI methodology, then the potential for Support II (the IPCT) to aid their Trusts/Boards would be greatly enhanced. At present, departments of health have tasked national data centres, e.g. HPS, HPE, with what is in effect performance monitoring. While it is still essential that Support III continues its epidemiology focus duties, changing from a performance-monitoring to a CQI approach could benefit all. Data produced by Support III are often released quarterly—a quarter behind; this is too slow for effective quality improvement. For optimal responses. the data interpretations must be available as close to real time as is possible (Benneyan, 1998). After significant major declines from the mid-2000s onwards in both MRSA BSI and CDI, it is now difficult to determine or monitor ongoing success from published national data. Although the major declines have not reversed, results have plateaued. Whether this is an indicator of falling IPC performance, increased challenges on the NHS system or changes in organism pathogenicity is unknown. The continued emergence of other pathogens (e.g. carbapenemase-producing *Enterobacteriaecae*), some of which are being attributed to healthcare, suggests that the discussed IPC improvements are insufficient and/or that the emerging pathogens are not the result of actions and inactions within care settings.

The evidence in this analysis suggests that national data producers should be charged with using a CQI approach when they feedback data and thus make it easy for those receiving the data to address issues in a timelier way. Numerical data, even at a national level, should be assessed for variation using the accepted CQI criteria. This variation should be communicated clearly. This would not only identify outliers in a negative but also positive sense and allow for, as stated, more timely responses to any identified deterioration of data and thus of systems themselves. Producing data in spreadsheets with only 12–13 points of data negates the possibility of a CQI assessment that requires 25 data points to conclude a process is in statistical control (Benneyan, 1998).

Although CDI and MRSA reductions were associated with the introduction of national targets, this was by no means a causal relationship (Wylie et al., 2011). New IPC performance indicators must plausibly relate to performance and be subject to reduction via system change. For *E. coli* bacteraemia (ECB), it is too soon to determine if success can be achieved by improved IPC. A recent review (Boswell et al., 2018) suggests that as few as 18% of ECBs may be preventable. While efforts must be made to prevent the preventable, the problem is that data are needed on an additional 82% of infections to find the 18% that might be preventable. There is also a question on the use of MSSA bacteraemia (used in Scotland) as an IPC performance indicator given its intransigence to reduction when compared to MRSA.

Healthcare is a complex system involving continuously changing people, environments, methods, equipment, micro-organisms and culture. Thus, any significant variation in numerical IPC performance indicators could be indicating success (or failure) in any of the component system parts that aid/reduce transmission or infection risk. Success could indicate IPC improvements and reduced surgical risks, but it could also indicate that micro-organisms have gained (or lost) some of their pathogenicity. Similarly, increasing microbial rates could indicate decreasing IPC performance and/or improved care with people living longer and eventually succumbing to an unpreventable infection in older age. Thus, the optimising of healthcare performance within a CQI framework (at all levels) and the analysing of data both epidemiologically and using a CQI model is essential. Although statistical monitoring will detect significant improvements (and deteriorations) in rates, SPCs are unable to attribute reasons for these changes. What can be said for certain is that healthcare environments have improved. However, in a healthcare system, ‘visibly clean’ may be insufficient given the known ability of pathogens to survive in a viable state for long periods in care environments. Continuous synergistic efforts at all levels of the organisation are needed to provide an IPC-safe environment and to enable the performance of IPC-safe procedures. Monitoring of systems quantitatively and qualitatively will always be needed and should always be subject to review and improvement.

## Limitations

The present study was an independent analysis of national priority documents and measures of success. Opinions were not sought from those who commissioned the documents or receive reports on the outputs and inspection reports; their views may have been different to those of the authors. Additionally, the analysis is written with the benefit of hindsight bias. That is, those commissioning the reports and seeking information on their implementation were essentially making decisions during uncertainty. At the time of commissioning, they could not be certain that their national priority documents were the solutions to the IPC problems. The project used publicly available data. There may be other data analyses available to commissioners which supports other conclusions.

## Conclusion

To reclaim the confidence of the general public in healthcare services, the extant priority documents have enabled an easy conclusion to Question 1: The local and national infection prevention and control priorities are evidenced by:

- people being cared for in an IPC-safe environment;
- staff following IPC-safe procedures;
- Trusts and boards continuously striving not just to attain standards, but to improve on them.

The qualitative indicators of IPC success are evidenced by:

- positive reports (not just by internal monitoring) of fit-for-purpose environments which are visibly clean and satisfy service users;
- an absence of negative media on IPC issues, e.g. increasing HAIs and/or visibly dirty care settings.

The quantitative indicators of IPC success are evidenced by:

- national reports of perceived nosocomial threats continuing to decline or at least failing to increase, e.g. CDI/MRSA and an absence of major outbreaks;
- SSI surveillance continuing to indicate a low incidence;
- the role of healthcare in their ongoing transmission of (re)-emerging pathogens being minimal or absent.

If national agencies that produce data were also charged with using a CQI model, then there would be further opportunities to detect and improve on successes. Furthermore, if national NHS authorities learnt from each other, there are opportunities to reduce redundancy and create a synergy of approach that could further reduce infection risks and the burden on those whose job is to keep people safe.

## Supplemental Material

sj-pptx-1-msj-10.1177_1757177420971849 – Supplemental material for A comparison of the nationally important infection prevention and control documents in NHS England and NHS Scotland Click here for additional data file. Supplemental material, sj-pptx-1-msj-10.1177_1757177420971849 for A comparison of the nationally important infection prevention and control documents in NHS England and NHS Scotland by Evonne T Curran, Emma Burnett, Jude Robinson and Heather Loveday in Journal of Infection Prevention
