# Supplementary material for: A comparison of the nationally important infection prevention and control documents in NHS England and NHS Scotland
Source: J Infect Prev. 2020 Nov 23;22(2):75–82. doi: 10.1177/1757177420971849 (PMC8014007; doi:10.1177/1757177420971849)
Supplement: sj-pptx-1-msj-10.1177_1757177420971849 – Supplemental material for A comparison of the nationally important infection prevention and control documents in NHS England and NHS Scotland [file sj-pptx-1-msj-10.1177_1757177420971849.pptx]

## Slide 1
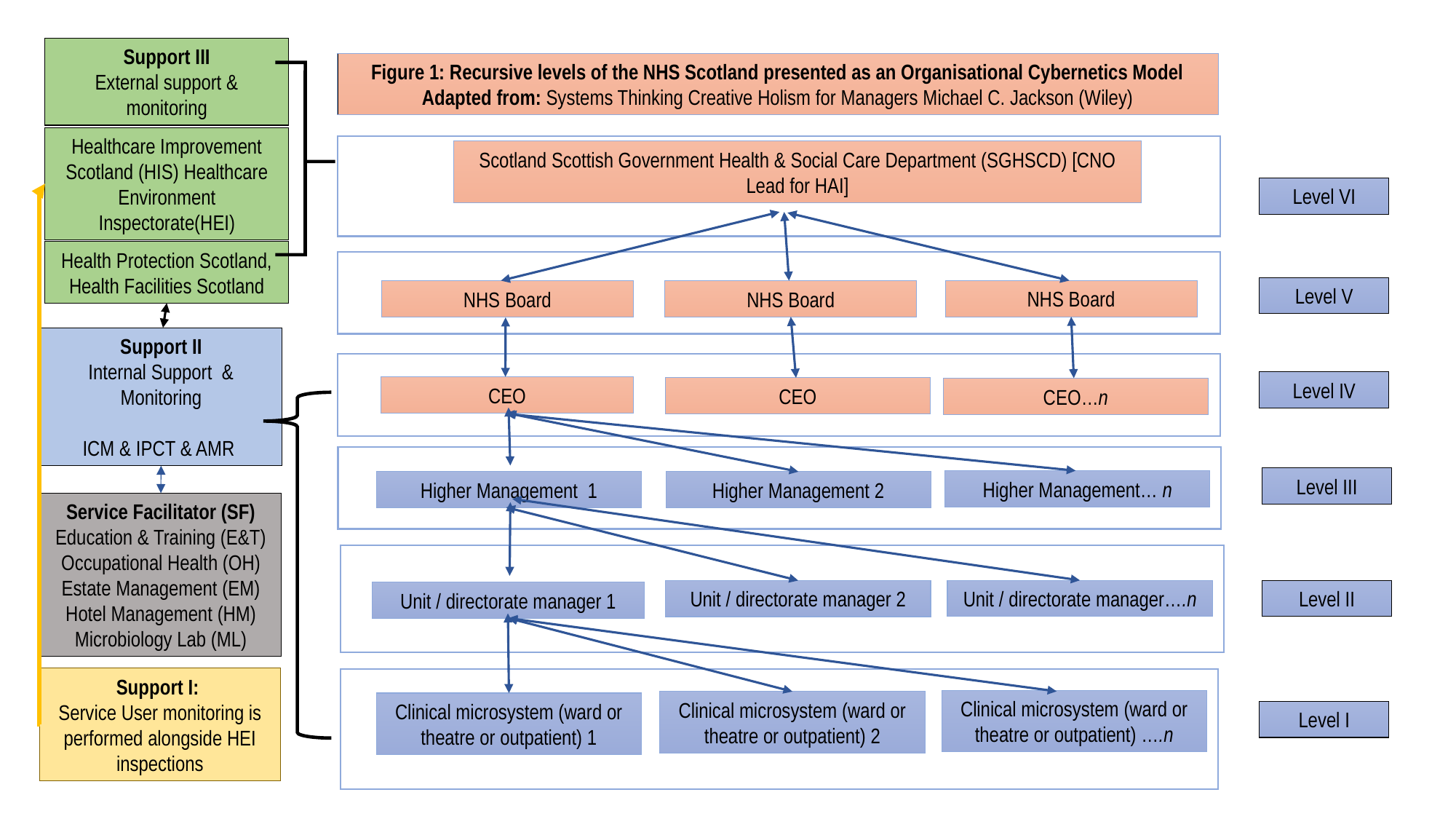

Support III
External support & monitoring
Figure 1: Recursive levels of the NHS Scotland presented as an Organisational Cybernetics Model
Adapted from: Systems Thinking Creative Holism for Managers Michael C. Jackson (Wiley)
Healthcare Improvement Scotland (HIS) Healthcare Environment Inspectorate(HEI)
Scotland Scottish Government Health & Social Care Department (SGHSCD) [CNO Lead for HAI]
Level VI
Health Protection Scotland, Health Facilities Scotland
NHS Board
NHS Board
NHS Board
Level V
Support II
Internal Support & Monitoring
ICM & IPCT & AMR
CEO
CEO
CEO…n
Level IV
Higher Management… n
Higher Management 1
Higher Management 2
Level III
Service Facilitator (SF)
Education & Training (E&T)
Occupational Health (OH)
Estate Management (EM)
Hotel Management (HM)
Microbiology Lab (ML)
Unit / directorate manager….n
Level II
Unit / directorate manager 2
Unit / directorate manager 1
Support I:
Service User monitoring is performed alongside HEI inspections
Clinical microsystem (ward or theatre or outpatient) ….n
Clinical microsystem (ward or theatre or outpatient) 2
Clinical microsystem (ward or theatre or outpatient) 1
Level I

## Slide 2
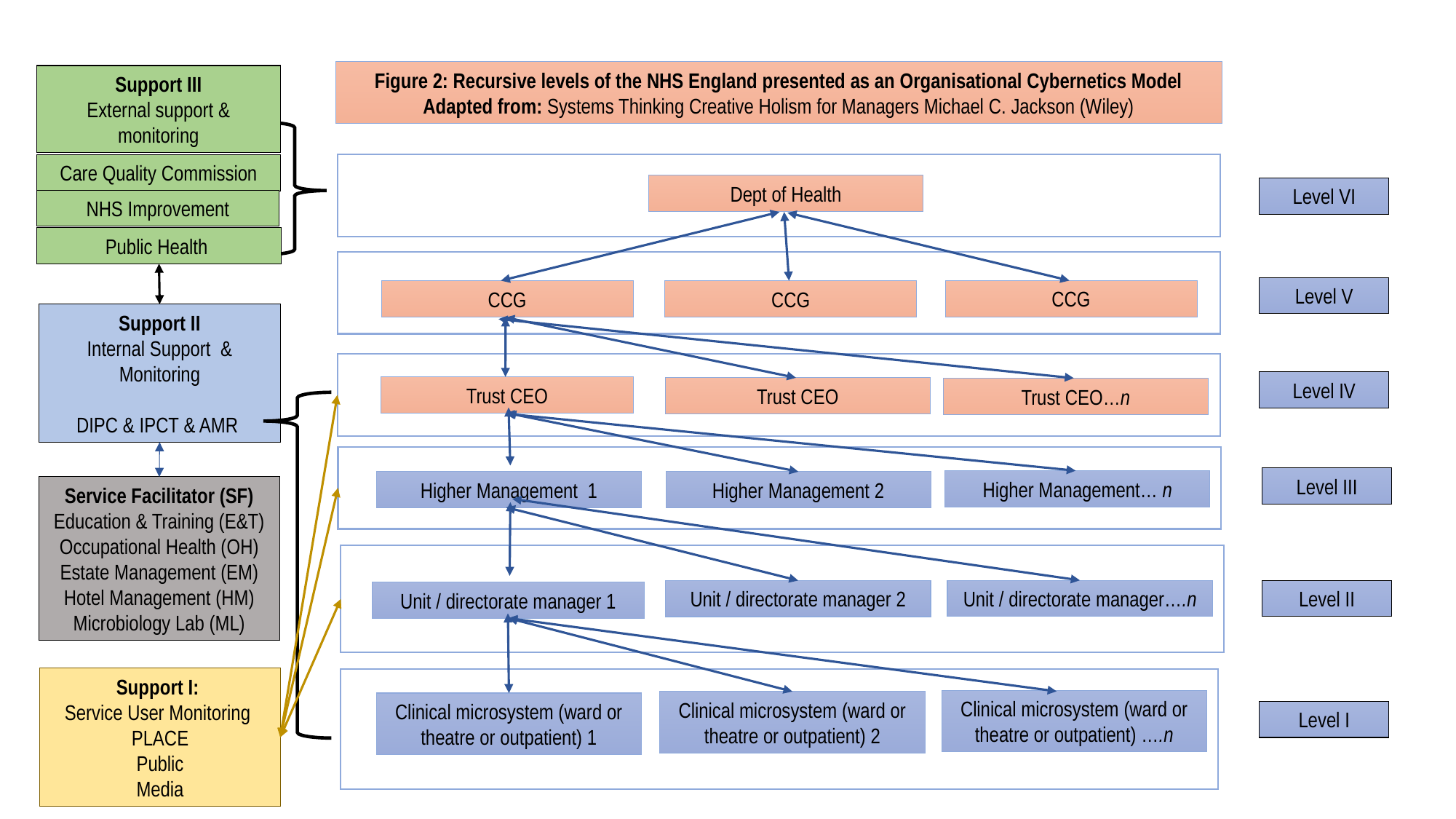

Figure 2: Recursive levels of the NHS England presented as an Organisational Cybernetics Model
Adapted from: Systems Thinking Creative Holism for Managers Michael C. Jackson (Wiley)
Support III
External support & monitoring
Dept of Health
Care Quality Commission
Level VI
NHS Improvement
Public Health
CCG
CCG
CCG
Level V
Support II
Internal Support & Monitoring
DIPC & IPCT & AMR
Trust CEO
Trust CEO
Trust CEO…n
Level IV
Higher Management… n
Higher Management 1
Higher Management 2
Level III
Service Facilitator (SF)
Education & Training (E&T)
Occupational Health (OH)
Estate Management (EM)
Hotel Management (HM)
Microbiology Lab (ML)
Unit / directorate manager….n
Level II
Unit / directorate manager 2
Unit / directorate manager 1
Support I:
Service User Monitoring
PLACE
Public
Media
Clinical microsystem (ward or theatre or outpatient) ….n
Clinical microsystem (ward or theatre or outpatient) 2
Clinical microsystem (ward or theatre or outpatient) 1
Level I

## Slide 3
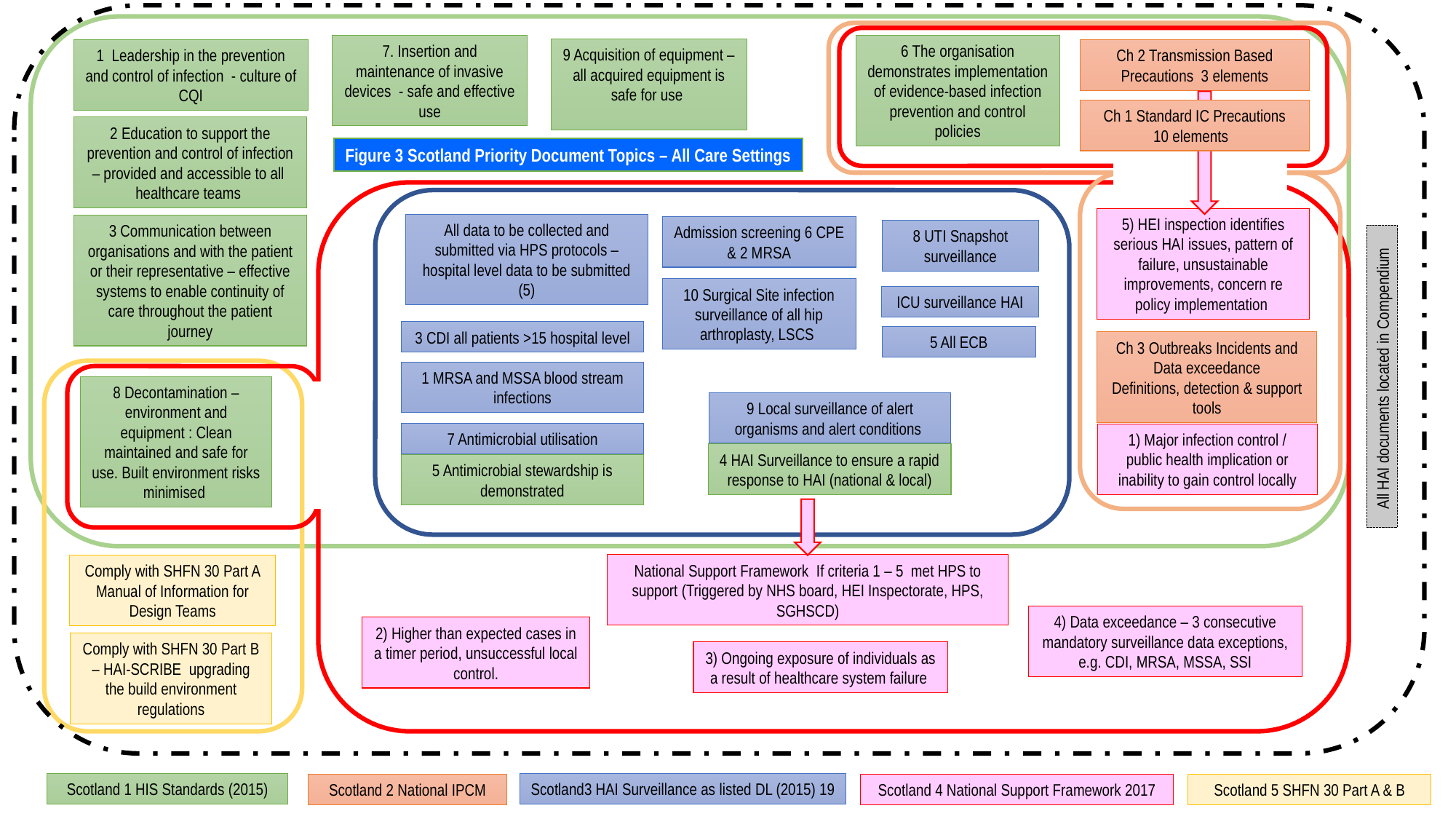

7. Insertion and maintenance of invasive devices - safe and effective use
6 The organisation demonstrates implementation of evidence-based infection prevention and control policies
9 Acquisition of equipment – all acquired equipment is safe for use
1 Leadership in the prevention and control of infection - culture of CQI
Ch 2 Transmission Based Precautions 3 elements
Ch 1 Standard IC Precautions
10 elements
2 Education to support the prevention and control of infection – provided and accessible to all healthcare teams
Figure 3 Scotland Priority Document Topics – All Care Settings
5) HEI inspection identifies serious HAI issues, pattern of failure, unsustainable improvements, concern re policy implementation
All data to be collected and submitted via HPS protocols – hospital level data to be submitted (5)
3 Communication between organisations and with the patient or their representative – effective systems to enable continuity of care throughout the patient journey
Admission screening 6 CPE & 2 MRSA
8 UTI Snapshot surveillance
10 Surgical Site infection surveillance of all hip arthroplasty, LSCS
ICU surveillance HAI
3 CDI all patients >15 hospital level
5 All ECB
Ch 3 Outbreaks Incidents and Data exceedance
Definitions, detection & support tools
All HAI documents located in Compendium
1 MRSA and MSSA blood stream infections
8 Decontamination – environment and equipment : Clean maintained and safe for use. Built environment risks minimised
9 Local surveillance of alert organisms and alert conditions
7 Antimicrobial utilisation
1) Major infection control / public health implication or inability to gain control locally
4 HAI Surveillance to ensure a rapid response to HAI (national & local)
5 Antimicrobial stewardship is demonstrated
National Support Framework If criteria 1 – 5 met HPS to support (Triggered by NHS board, HEI Inspectorate, HPS, SGHSCD)
Comply with SHFN 30 Part A Manual of Information for Design Teams
4) Data exceedance – 3 consecutive mandatory surveillance data exceptions, e.g. CDI, MRSA, MSSA, SSI
2) Higher than expected cases in a timer period, unsuccessful local control.
Comply with SHFN 30 Part B – HAI-SCRIBE upgrading the build environment regulations
3) Ongoing exposure of individuals as a result of healthcare system failure
Scotland 1 HIS Standards (2015)
Scotland3 HAI Surveillance as listed DL (2015) 19
Scotland 2 National IPCM
Scotland 4 National Support Framework 2017
Scotland 5 SHFN 30 Part A & B

## Slide 4
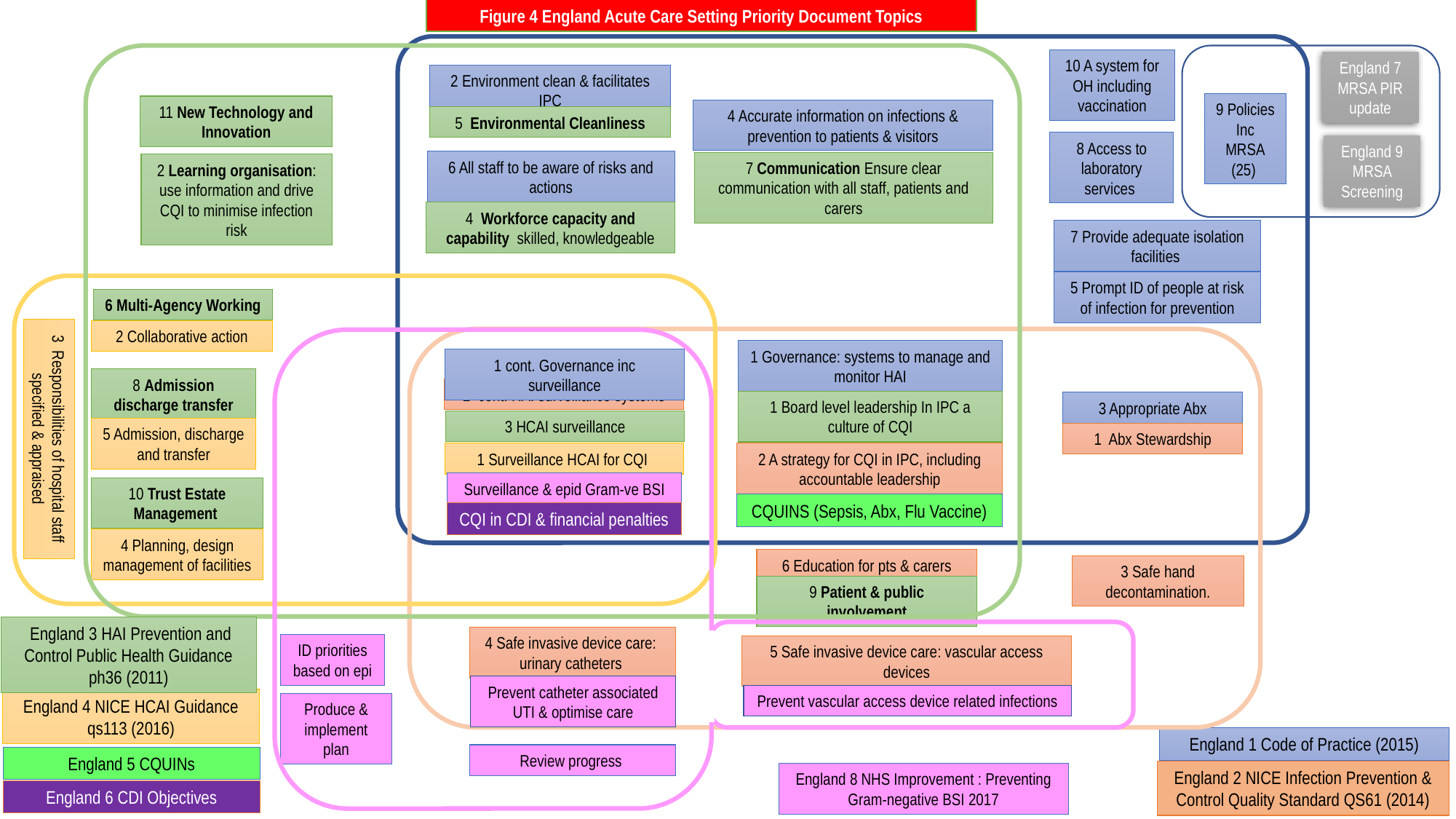

Figure 4 England Acute Care Setting Priority Document Topics
10 A system for OH including vaccination
England 7
MRSA PIR update
2 Environment clean & facilitates IPC
9 Policies Inc
MRSA
(25)
11 New Technology and Innovation
4 Accurate information on infections & prevention to patients & visitors
5 Environmental Cleanliness
8 Access to laboratory services
England 9
MRSA Screening
6 All staff to be aware of risks and actions
7 Communication Ensure clear communication with all staff, patients and carers
2 Learning organisation: use information and drive CQI to minimise infection risk
4 Workforce capacity and capability skilled, knowledgeable
7 Provide adequate isolation facilities
5 Prompt ID of people at risk of infection for prevention
6 Multi-Agency Working
2 Collaborative action
1 Governance: systems to manage and monitor HAI
1 cont. Governance inc surveillance
8 Admission discharge transfer
2 cont. HAI surveillance systems
1 Board level leadership In IPC a culture of CQI
3 Appropriate Abx
3 HCAI surveillance
3 Responsibilities of hospital staff specified & appraised
5 Admission, discharge and transfer
1 Abx Stewardship
1 Surveillance HCAI for CQI
2 A strategy for CQI in IPC, including accountable leadership
Surveillance & epid Gram-ve BSI
10 Trust Estate Management
CQUINS (Sepsis, Abx, Flu Vaccine)
CQI in CDI & financial penalties
4 Planning, design management of facilities
6 Education for pts & carers
3 Safe hand decontamination.
9 Patient & public involvement
 England 3 HAI Prevention and Control Public Health Guidance ph36 (2011)
4 Safe invasive device care: urinary catheters
ID priorities based on epi
5 Safe invasive device care: vascular access devices
Prevent catheter associated UTI & optimise care
Prevent vascular access device related infections
England 4 NICE HCAI Guidance qs113 (2016)
Produce & implement plan
England 1 Code of Practice (2015)
Review progress
England 5 CQUINs
England 2 NICE Infection Prevention & Control Quality Standard QS61 (2014)
England 8 NHS Improvement : Preventing Gram-negative BSI 2017
England 6 CDI Objectives

## Slide 5
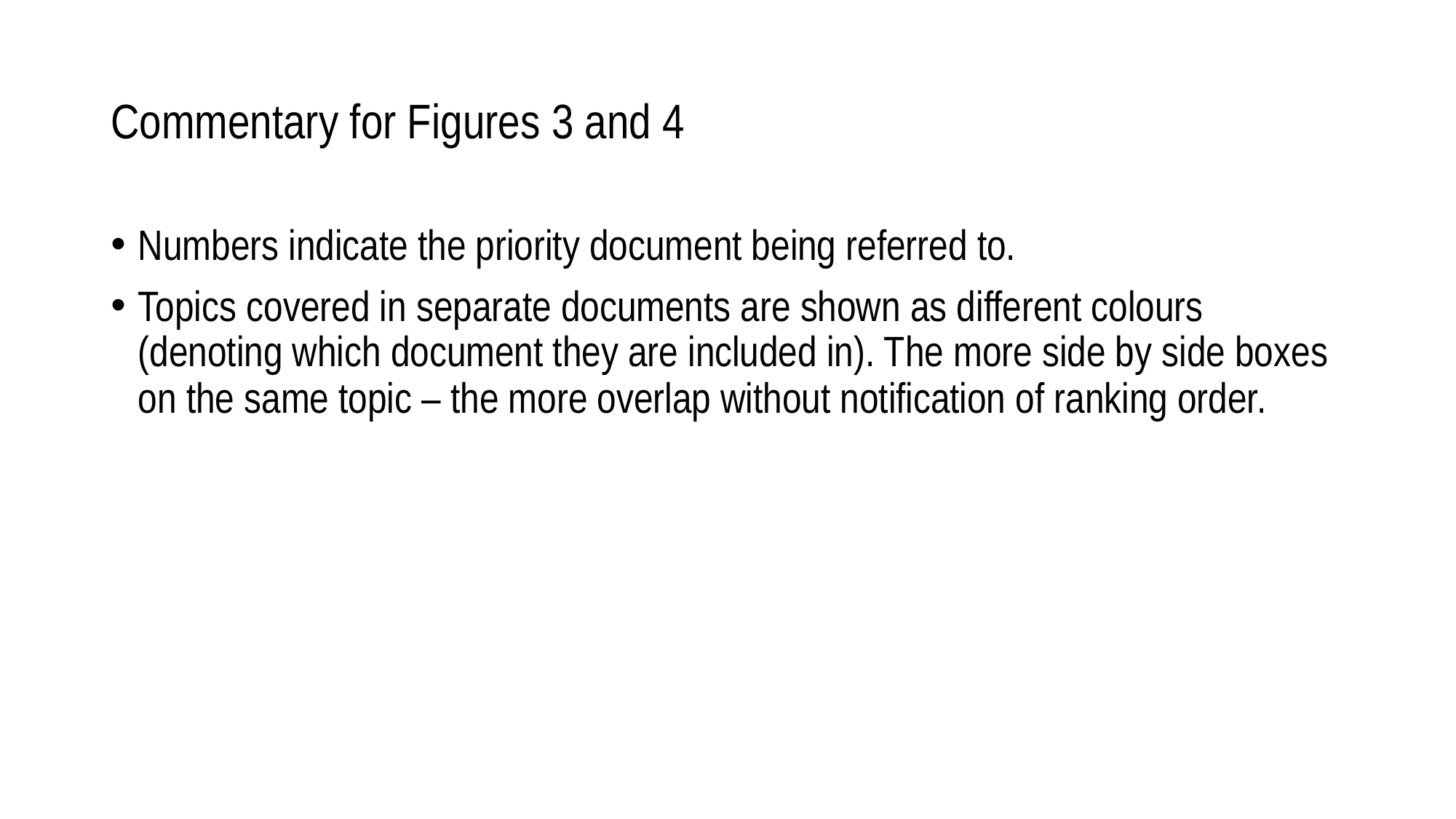

# Commentary for Figures 3 and 4
Numbers indicate the priority document being referred to.
Topics covered in separate documents are shown as different colours (denoting which document they are included in). The more side by side boxes on the same topic – the more overlap without notification of ranking order.
